# The contribution of gut bacterial metabolites in the human immune signaling pathway of non-communicable diseases

**DOI:** 10.1080/19490976.2021.1882927

**Published:** 2021-02-16

**Authors:** F. Hosseinkhani, A. Heinken, I. Thiele, P. W. Lindenburg, A. C. Harms, T. Hankemeier

**Affiliations:** a Division of Systems Biomedicine and Pharmacology, Leiden Academic Centre for Drug Research, Leiden University, Leiden, Netherlands; b Division of System Biomedicine, College of Medicine, Nursing and Health Sciences, National University of Ireland, Galway, Ireland; c Research Group Metabolomics, Faculty Science & Technology, Leiden Centre for Applied Bioscience, University of Applied Sciences, Leiden, Netherlands

**Keywords:** Bacterial metabolites, immune signaling, non-communicable diseases, gut microbiota, metabolomics, system biology

## Abstract

The interaction disorder between gut microbiota and its host has been documented in different non-communicable diseases (NCDs) such as metabolic syndrome, neurodegenerative disease, and autoimmune disease. The majority of these altered interactions arise through metabolic cross-talk between gut microbiota and host immune system, inducing a low-grade chronic inflammation that characterizes all NCDs. In this review, we discuss the contribution of bacterial metabolites to immune signaling pathways involved in NCDs. We then review recent advances that aid to rationally design microbial therapeutics. A deeper understanding of these intersections between host and gut microbiota metabolism using metabolomics-based system biology platform promises to reveal the fundamental mechanisms that drive metabolic predispositions to disease and suggest new avenues to use microbial therapeutic opportunities for NCDs treatment and prevention.

**Abbreviations**: NCDs: non-communicable disease, IBD: inflammatory bowel disease, IL: interleukin, T2D: type 2 diabetes, SCFAs: short-chain fatty acids, HDAC: histone deacetylases, GPCR: G-protein coupled receptors, 5-HT: 5-hydroxytryptamine receptor signaling, DCs: dendritic cells, IECs: intestinal epithelial cells, T-reg: T regulatory cell, NF-κB: nuclear factor κB, TNF-α: tumor necrosis factor alpha, Th: T helper cell, CNS: central nervous system, ECs: enterochromaffin cells, NSAIDs: non-steroidal anti-inflammatory drugs, AhR: aryl hydrocarbon receptor, IDO: indoleamine 2,3-dioxygenase, QUIN: quinolinic acid, PC: phosphatidylcholine, TMA: trimethylamine, TMAO: trimethylamine *N*-oxide, CVD: cardiovascular disease, NASH: nonalcoholic steatohepatitis, BAs: bile acids, FXR: farnesoid X receptor, CDCA: chenodeoxycholic acid, DCA: deoxycholic acid, LCA: lithocholic acid, UDCA: ursodeoxycholic acid, CB: cannabinoid receptor, COBRA: constraint-based reconstruction and analysis

## Introduction

In the past century, a disturbing trend has been the consistent rise in non-communicable diseases (NCDs) which currently represent 70% of global mortality.^1^ NCDs are chronic diseases that have in common an underlying low-grade inflammation. These include early-onset NCDs, such as allergies, asthma, and some autoimmune diseases, and later-onset NCDs, including cardiovascular disease (CVD), metabolic syndrome, and neurodegenerative disorders.^1,2^ Under normal conditions, inflammation induces a physiological response to keep homeostatic balance within tissues. However, the chronic inflammatory states that are observed in NCDs do not appear to fit this model as tissues adapt to new physiological or metabolic conditions. Although inflammation and the pathways to disease are multifactorial, extensive application of next-generation sequencing and gnotobiotics technologies (germ-free conditions or when inoculated with known microorganisms) have revealed increased implication of altered gut colonization patterns (dysbiosis) in the physiologic, immunologic, and metabolic dysregulation, seen in a myriad of NCDs (Table 1). ^22,23^ For example, gut metagenomic profiles of patients with inflammatory bowel disease (IBD) are highly correlated with fecal calprotectin levels, a biomarker for severity of inflammation in IBD.^15^ Moreover, a significant correlation between gut microbiota dysbiosis and an elevated level of interleukin 6 (IL-6) was found in plasma samples of patients with type 2 diabetes (T2D).^24^

**Table 1.** Impact of gut microbiota on immune system modulation in non-communicable diseases

Pathology type		Bacterial dysbiosis	Altered bacterial metabolites
**Neurodegenerative diseases**	AD	^3,4^ **↑** *Blautia, Bacteroides* **↓***Firmicutes, Bifidobacterium, Dialister*	^5,6^ SCFAs, Serotonin, indole and indole acid derivatives Kynurenine, BAs, vitamin B
	PD	^3,7^ **↑** *Blautia, Ralstonia, Lactobacillus* spp., Proteobacteria **↓** *Bacteroides, Prevotella*, *Faecalibacterium*	^5,7,8^ SCFAs, Serotonin, Kynurenine, BAs, vitamin B
	MS	^3,4^ **↑** *Pseudomonas*, *Mycoplana, Haemophilus, Blautia* **↓** *Bacteroides*, *Faecalibacterium*, Clostridia clusters XIVa and IV	^5,9^ SCFAs, Serotonin, Kynurenine, indole acid derivatives, vitamin B
**Metabolic syndrome**	Type II diabetes	^10^ **↑** *Lactobacilllus* spp., *Bifidobacterium* spp., *Enterococcus* spp. **↓** *Akkermansia muciniphila, Clostridium leptum, Enterobacter* spp.	^10–12^ SCFAs, BAs, vitamin B12
	NAFLD	^13,14^ **↑** *Firmicutes, Clostridium coccoides, Escherichia coli* **↓***Bacteroides, Prevotella*	^13,14^ BAs phenolic acids
**Allergic and immune disorders**	IBD	^15,16^ **↑** *Escherichia coli, Veillonella* spp., *Rumnicoccus* spp. **↓***Faecailbacterium prausnitzii, Lactobacillus* spp. *Bifidobacterium* spp.	^15–17^ SCFAs, B vitamin, Bas, N1,N12-diacetylspermine, indole, Kynurenine
	Asthma	^18,19^ **↑** Bacteroidetes, *Clostridium difficile* **↓***Bifidobacteria, Actinobacteria, Veillonella* spp.,*Faecalibacterium, Rothia* spp.	^18,20,21^ SCFAs, BAs, phenolic acids

The human gut is a diverse niche which hosts hundreds of species of commensal bacteria, protozoa, and fungi that live in a symbiotic relationship with their host and has a wide-ranging impact on host physiology.^25^ Accordingly, the preservation of beneficial interactions between the host and its gut microbiota is a key requirement for health. Although the enigmatic network of interactions is beginning to be unraveled, inflammation as a consequence of dysbiosis indicates the importance of immune signaling in communication between the host and gut microbiota.^26^ Considering that the gut is the residence for up to 80% of immune cells in the body and that microbiota contain nearly 150-fold more genes than the host genome, it is not surprising that bacterial metabolites play an important role in educating immune cells and modulating immune signals.^27,28^ Engagement of gut microbial metabolites with host immune receptors both locally and on extra-intestinal organs form an extensive array of signals that respond to changes in the environment, nutrition, health, and immunological status. In turn, the immune system participates in shaping and preserving the ecology of the gut microbiota.^28,29^ Therefore, in individuals with dysbiosis, disturbance of immune signaling pathways can lead to chronic, non-resolving inflammation, and tissue damage which has emerged as a common feature in NCDs.^1^

Despite a large amount of metagenomics data on the role of gut microbiota dysbiosis in the development of NCDs, the underlying mechanisms in which bacterial metabolites influence the host immune system remain to be fully elucidated. A deeper understanding of the intersections between host and gut microbiota metabolism promises to reveal the fundamental mechanisms that drive metabolic predispositions to disease and might suggest new metabolite-based therapeutic opportunities for NCDs. Mechanistic models that predict the potential of individual microbes to process certain metabolites could aid in revealing microbe-metabolite-receptor associations and improve our understanding of the effect of a specific microbial community on the metabolome and immune signaling. In this review, we provide an overview of gut bacterial metabolites in NCDs with a physiological effect on major immune functions. We then review recent advances that help our ability to rationally design microbial therapeutics.

## Microbiota and gut metabolites

One of the essential functions of the gut microbiota comprises the modulation of gastrointestinal metabolites, including their synthesis, digestion, fermentation, and secondary metabolism. It has been estimated that over 8000 non-nutritious compounds (such as dietary fiber and polyphenols that are not classified within the six basic nutrient groups) are present in the human gut, of which most compounds are not digested by human digestive enzymes.^30^ Macronutrients (lipids, carbohydrate and proteins) and complex dietary fibers that reach the colon, undergo microbial fermentation and catabolism, resulting in the production of essential metabolites such as short-chain fatty acids (SCFAs), essential amino acids, vitamins, and hormones. Although the colon is the major site of this fermentation, the microbiota at other sites, such as small intestine (mainly lactobacilli and streptococci), contribute to regulation of nutrient absorption and metabolism conducted by the host.^31^ In gut microbiota dysbiosis, numerous of microbial metabolites are altered. According to the type and concentration of metabolites, host sensing receptors respond.^32^ In the following sections, we will highlight key examples of gut bacterial metabolites and how these metabolite shifts contribute to the downstream modulation of host immunity and chronic inflammation in NCDs.

## Short-chain fatty acids

SCFAs are among the well-studied metabolites because of their important role in human health.^33^ SCFAs (mainly butyrate, propionate, and acetate) are present in high concentrations (20–140 mM per day depending on the diet) in the human cecum and the ascending and transverse colon as end products of bacterial fermentation of dietary fiber.^28,34,35^ Due to the lack of polysaccharide lyase enzymes in humans, undigested dietary fiber reaches the colon where it undergoes a two-stage degradation. The degradation is done mainly by bacterial genera such as *Bacteroides, Clostridium, Bifidobacterium, Prevotella*, and *Ruminococcus*.^35^ In addition to their role as an important energy source (have been extensively reviewed elsewhere^36,37^), SCFAs are involved in a broad array of double-faced functions that influence both host immune signaling and the commensal microbial community.^33,38^ A considerable amount of studies highlight the importance of SCFAs for immune system functioning. For instance, Nagpal *et al*.^39^ showed that application of infant gut-origin *Lactobacillus* and *Enterococcus* strains increase the level of SCFAs in feces and moderate the gut microbiota perturbation resulting in a reduction of inflammation in both mouse and human models. Furthermore, they revealed that the antagonistic activity of these strains against clinical isolates of uropathogenic *E. coli* and *Klebsiella pneumonia* was due to the production of SCFAs.

**Figure 1.**
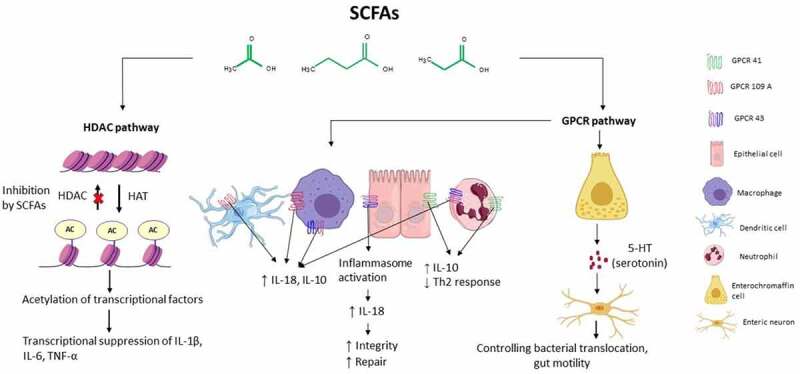
Immune signaling pathway activated by Short-Chain Fatty Acids. SCFAs act on immune cells mainly through two pathways: activation of G-protein coupled receptors (GPCRs) such as GPCR 41, GPCR 43, and GPCR 109 and inhibition of histone deacetylase (HDAC). AC: acetylate; HAT: histone acetylase

SCFAs can modulate immune cells by affecting their cytolytic activity, cytokine production, and regulation of gene expression.^40^ The anti-inflammatory effect of SCFAs are mediated through two major signaling pathways: 1) inhibition of histone deacetylases (HDAC), and 2) activation of G-protein-coupled receptor signaling (GPCR) (Figure 1). ^26,33^

1. HDAC inhibition

HDAC activity can decrease histone acetylation and thereby modulate gene expression in a variety of cells. Inhibition of HDAC can result in increased expression of some genes, but may at the same time induce acetylation of some transcription factors and further suppression of the transcription process.^41^ As an HDAC inhibitor, SCFAs can inhibit inflammation by suppressing the expression of the pro-inflammatory cytokines interleukin 6 (IL-6) and IL-12 in various immune cells such as local macrophages and dendritic cells (DCs).^26^ IL-6 is among the major factors that mediate inflammation into its chronic phase. Elevated levels of IL-6 in the brain have been observed in different neurodegenerative diseases such as multiple sclerosis, Parkinson’s and Alzheimer’s disease.^42^ An increased plasma level of IL-6 has also been reported in patients with T2D, which was in conjunction by a prevalence of *Prevotella copri* and *Bacteroides vulgatus* in their feces.^24^ Butyrate is able to regulate the transforming growth factor β promoter in intestinal epithelial cells (IECs) in an HDAC-dependent manner leading to the accumulation of T-regulatory (T-reg) cells in the gut.^43^ Colonic T-reg cells are critical for limiting intestinal inflammation. Interestingly, a beneficial effect of butyrate is associated with a reduction of nuclear factor κB (NF-κB) translocation in macrophages within the lamina propria of patients with Crohn’s disease.^20^

HDAC inhibition by SCFAs is not restricted to immune cells in the gut. SCFAs can be transported across the gut epithelium into the circulation, affecting distant organs. For instance, exposure of peripheral blood mononuclear cells and neutrophils to SCFAs *in vitro* can reduce NF-κB activity via HDAC inhibition and thereby down-regulate production of the pro-inflammatory cytokine tumor necrosis factor alpha (TNF-α).^35^ In the lungs, acetate and propionate control the hyperactivity of airways by inhibiting HDAC and reducing the action of T helper cell (Th-2) by increasing DC proliferation.^18^ Maternal acetate during pregnancy can protect the offspring against allergic airway disease,^44^ whereas HDAC inhibitors such as sodium butyrate markedly inhibit brain microglia-induced inflammation.^45^ Thus, the HDAC pathway actively contributes to the maintenance of immunological tolerance and control of pro-inflammatory cytokines.

1. GPCR activation

GPCRs are one of the largest and most diverse families of integral membrane protein receptors in mammalian genomes. Many signaling pathways in the human body rely on this family of receptors and therefore play an important role in medicine and drug development.^46^ A high presence of GPCRs is found within the gastrointestinal tract (mainly GPCR41, GPCR43, and GPCR109), emphasizing the importance of this receptor family for the regulation of host-microbial interactions.^47^ Butyrate can bind GPCR109 on epithelial cells, macrophages, and DCs and trigger the secretion of IL-18 and IL-10, which further leads to suppression of inflammation. Deficiency of IL-10 in human and animal models leads to intestinal inflammation and the growth of enteric pathogens which can promote the development of colorectal cancer.^46^ GPCR43 is present in different tissues such as adipose tissue and gastrointestinal cells, with the highest expression found in immune cells.^48^ Recent evidence indicates that a high-fiber diet activates GPCR43 on IECs, which results in activation and assembly of the inflammasome, a protein complex that enables maturation of the pro-inflammatory cytokines by caspase-1-mediated cleavage. Formation of inflammasomes leads to downstream production of IL-18, a key cytokine for the repair and maintenance of the epithelial integrity.^49^ However, in the context of colitis, ambivalent results have been published, which presents a possible dual role of SCFAs and GPCR43 in controlling inflammation.^50^ This explains the importance of cell types and their location for the downstream activation of GPR43.

The interaction of SCFAs with GPCRs are not limited to the gut. Maturation and proper functioning of murine microglia in the central nervous system (CNS) is dependent on SCFAs and GPCR43.^51^ Moreover, activation of GPCR41 in enteric nerves, mainly by butyrate and propionate, indicates that SCFAs signaling may be transferred directly to the nervous system.^52^ Activation of GPCR41 signaling by SCFAs was also shown to protect against allergic airway disease by reducing Th2 cell activity in the lungs.^18^ Although SCFA-mediated GPCR signaling has been shown to play an important role in modulating the host immune system, it remains an extremely complex process which is still at an early stage of investigation.

Preclinical animal studies suggest that SCFAs can influence gastrointestinal motility. Immune cells express a wide range of GPCRs as neurotransmitter receptors, which can respond to serotonin modulation. Enterochromaffin cells (ECs) are a type of chemosensory cells within the gut epithelium and are involved in neural monitoring of the gut. ECs can affect gastrointestinal motility and secretion through serotonin production.^28^ ECs synthesize more than 90% of the serotonin in the human body. SCFAs can promote the production of serotonin in the enteric nervous system via GPCR 43 and GPCR 41, and affect gut motility through 5-HT signaling (5-hydroxytryptamine receptor signaling). Moreover, blocking 5-HT signaling results in bacterial translocation across the intestinal barrier and inflammation.^27^ Gastrointestinal motility disorders are frequent in patients with Parkinson’s disease, multiple sclerosis, IBD.^53–55^

Another way in which SCFAs affect the immune system is epigenetic regulation of gene expression. One such example is the butyrate-induced up-regulation of *muc* genes and mucus secretion in goblet cells, which is essential for mucosal immunity and gastrointestinal integrity.^56^

Although many studies discussed the importance of SCFAs for human health, the interaction between SCFAs and their receptors might not always be beneficial to the host. For instance, butyrate induced aberrant proliferation and transformation of colon epithelial cells in a genetically susceptible mouse model of colorectal cancer.^57^ Moreover, SCFAs can aggravate neutrophilic inflammatory response, promoting the outgrowth of *Pseudomonas aeruginosa* in patients with cystic fibrosis.^58^ Therefore, further research on the immunomodulatory effects of SCFAs based on concentration and target cells is warranted.

In addition to modulating the immune system, SCFAs can be important in gut bacterial communication. A low fiber and/or high-fat diet can remarkably reduce gut microbiota diversity, exemplified by abnormally high Firmicutes and low Bacteroidetes abundances in multiple NCDs.^59^ Inoculation of SCFAs producing bacteria can increase Bacteroidetes abundances while reducing Firmicutes, and improve balance in the gut bacterial structure.^34^

Overall, these data have led to emerging interests in the regulatory function of SCFAs in a wide range of immune cells, including both innate and adaptive cells. The effect of SCFAs, whether on the immune system or gut microbial community, is concentration-dependent and the favorable normal physiological range needs further research, as discussed below. Understanding the interaction of SCFAs with their receptors in detail might provide great insights into the development of new therapeutic avenues to treating NCDs patients through dietary manipulation.

## Indole and indole acid derivatives

Amino acid metabolism has an important impact on immune cell function. Although endogenous metabolism of amino acids by human play important roles in regulating gut immune function, the potential contribution of resident microbiota should not be ignored. Amino acids that reach the lower part of the gut are transformed by specific bacteria, resulting in the production of specific amino acids with signaling properties. The most abundant amino acid fermenting bacteria in humans belong to the *Clostridium* clusters, the *Bacillus-Lactobacillus-Streptococcus* groups, and *Proteobacteria*.^60^

**Figure 2.**
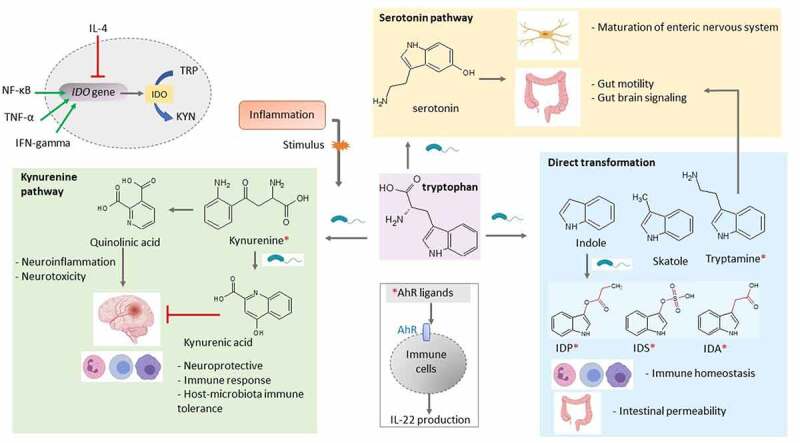
Overview of the different ways microbes degrade tryptophan in the human gut. Tryptophan metabolites regulate various host processes through their function as signaling molecules. In the gut, tryptophan can undergo several possible chemical alterations: the direct transformation of tryptophan by the gut microbiota into several molecules, such as indole and its derivatives, and indirectly through the kynurenine pathway and the serotonin pathway. Various tryptophan catabolites are ligands for the aryl hydrocarbon receptor (AhR) expressed on intestinal immune cells and thereby alter innate and adaptive immune responses. AhR ligands are denoted with a red asterisk. Pro-inflammatory factors (NF-κB, TNF-α and interferon gamma) upregulate indoleamine 2,3-dioxygenase (*IDO*) expression, whereas anti-inflammatory factors such as IL-4 inhibit its expression. IDP: indole propionic acid; IDS, indoxyl sulfate, IDA: indoxyl acetic acid

L-tryptophan is an aromatic amino acid composed of a β carbon connected to the 3-position of an indole group. In the human body, several signaling molecules are derived from tryptophan through three major pathways: kynurenine pathway, the serotonin pathway or direct transformation to different indole derivatives compounds such as tryptamine, indole and its derivatives.^28,61,62^ The physiological effect of compounds derived from tryptophan metabolism can be either protective or detrimental depending on the signaling pathway and target tissue involved (Figure 2).

Indole is one of the major products of bacterial tryptophan metabolism. It has been detected in different concentrations (0.25–1.1 mM) in feces of healthy individuals, while a significant decrease (3.5 × 10^−7^ – 0.00110 μM/g feces) has been reported in different NCDs such as IBD, autoimmunity, neurodegeneration and diabetes.^28,63,64^ Exposure of human colon-cancer cell line to 1 mM indole *in vitro* could inhibit inflammation by up-regulating the expression of the anti-inflammatory cytokine IL-10, decreasing TNF-α-mediated activation of NF-κB, and expression of the pro-inflammatory chemokine IL-8.^65^ Furthermore, indole promotes epithelial barrier functions of intestinal cells by inducing increased expression of tight junction proteins between cells, thereby contributing to trans epithelial resistance to inflammation.^62^ Additionally, indole administration attenuated multiple deleterious effects of non-steroidal anti-inflammatory drugs (NSAIDs) on the gut in a murine model of NSAID enteropathy, by modulating neutrophilic inflammation and altering the gut microbiota composition and diversity.^66^ Together, these studies highlight the anti-inflammatory and beneficial effects of indole on intestinal health.

Indole-3-aldehyde and indole-3-propionic acid are indole derivatives that can act as ligands for the aryl hydrocarbon receptor (AhR).^67^ AhR is a member of the ligand-activated transcription factor family on epithelial and immune cells and a powerful modulator of immune cell proliferation.^9^ Zelante et al.^61^ showed that production of Indole-3-aldehyde by a subset of commensal lactobacilli in small intestine such as *L. reuteri* and *L. johnsonii* activate innate lymphoid cells to release IL-22, resulting in secretion of anti-microbial peptides by epithelial cells, mucosal protection from inflammation, and fortification of epithelial cell tight junctions. In mouse models, decrement of the gut microbiota using the antibiotic ampicillin decreased the levels of the AhR agonist indoxyl-3-sulfate and worsened autoimmune encephalomyelitis disease scores.^68^ In human astrocytes, indoxyl-3-sulfate treatment reduces the expression of pro-inflammatory factors such as TNF- α and IL-6. Supplementation with the tryptophan metabolites indoxyl-3-sulfate, indole-3-propionic acid and indole-3-aldehyde was successful in reducing CNS inflammation through AhR in autoimmune encephalitis mouse models.^61^ Moreover, a recent study by Ageuro et al.^69^ indicated that indole derivatives from gestationally-colonized maternal microbiota increased mononuclear phagocytes in their offspring.

Skatole (3-methylindole) is another indole-derived metabolite that occurs naturally in feces. The fecal skatole concentration can vary considerably between individuals and may indicate different health states.^65^ Fecal skatole levels in healthy individuals are usually ~5 μg/g feces, whereas fecal skatole levels may increase up to 80–100 μg/g feces in persons who suffer from disturbed intestinal microbiota, such as patients with colorectal and colon cancer.^70^ Despite little information is available on the role of skatole in immune signaling, an elevated level in inflammation may highlight the importance of further studies.

The tryptophan-derived monoamines, tryptamine, and serotonin, stimulate gut peristalsis by signaling through the serotonin receptors in the gut, as discussed above.

Tryptophan metabolism through the kynurenine pathway is mediated by either indoleamine 2,3-dioxygenase (IDO) or tryptophan 2,3-dioxygenase which leads to the production of kynurenine and downstream products, such as kynurenic acid and quinolinic acid (QUIN). The gut microbiota is known for its contribution to the kynurenine pathway by regulating IDO 1 expression.^61,71^ Although IDO-dependent tryptophan metabolism plays a minor role under normal conditions, it is strongly upregulated in response to inflammatory factors (e.g., interferon gamma, TNF-α and NF-κB).^17,72^ Overexpression of IDO 1 and increased QUIN levels have been reported in IBD patients and different neurological disorders such as Alzheimer’s disease, anxiety, depression, epilepsy and human immunodeficiency virus-associated neurocognitive disorders.^73^ Gut bacteria such as *Lactobacillus* spp., *Pseudomonas aeruginosa, Pseudomonas fluorescens* possess the enzyme aspartate aminotransferase, and produce kynurenic acid by transamination of kynurenine.^72^ Increased level of kynurenic acid has been reported in patients suffering from IBD, T2D, and multiple sclerosis.^74^ Kynurenic acid can modulate the intestinal inflammation and limit inflammation-induced cell damage in CNS through GPCR35 and AhR mediated signals. Moreover, kynurenic acid can increase energy expenditure by activating GPCR35, which stimulates lipid metabolism and anti-inflammatory phenotype in adipose tissue.^75^

As yet, the characterization of tryptophan-metabolizing organisms, together with their associated biochemical pathways, remains an indispensable challenge. Development of high-throughput and high-sensitivity methods, as well as the integration of multi-omics modalities, offer promising strategies to identify the variety of microorganisms and microbial genes involved in the modulation of tryptophan metabolism.

## Choline

Choline is an essential dietary nutrient for humans and is required for the synthesis of the neurotransmitter acetylcholine, the membrane lipid phosphatidylcholine (PC) and sphingomyelin, and the methyl donor glycine betaine. Previous work unraveled a critical role for gut microorganisms (*Firmicutes*; especially the *Clostridium* cluster XIVa and *Eubacterium, Actinobacteria* and *Proteobacteria*) in the degradation of choline to trimethylamine (TMA), which is further metabolized in the liver to trimethylamine *N*-oxide (TMAO). Systemic concentration of TMAO in normal healthy individuals ranges from 0.5 to 5 μM and can vary greatly after the intake of certain foods.^76,77^

It is important to highlight that less than 1% of the gut microbiota harbor the genes required for TMA production. Nevertheless, even very low concentrations of these gut bacteria seem to be sufficient for TMA production, which illustrates the importance of the gut microbiota in this context. Recently, Chittim et al.^78^ explored the origin of the choline metabolized by the gut microbiota and discovered that choline-utilizing gut microorganisms can hydrolyze PC using a phospholipase D enzyme and further convert the released choline to TMA. This finding provides novel opportunities for treating TMA-related pathologies, as altered plasma levels of TMAO have been implicated in a number of adverse host pathologies, including CVD, nonalcoholic steatohepatitis (NASH), IBD and CNS disorders. Choline-deficient diets and subsequent low serum TMAO levels have been associated with NASH, by reducing the microbiota diversity and inducing intestinal dysbiosis. For instance, choline deficient diets induced a remarkable decrease in abundance of amongst others *Bifidobacteria* and *Lactobacilli*, whereas microbial clades such as *Bacteriodetes, Ruminococus* and *Firmicutes* were increased.^79,80^ It is suggested that this dysregulation in the microbiota promotes NASH development by activating the inflammasome, as inflammasome activity is involved in formation of fibrotic plaques in NASH pathology.^81^ These data indicate that choline is necessary for maintaining a healthy gut microbiota. Moreover, choline has been shown to exert anti-inflammatory effects. For example, in asthma patients, supplementation of choline significantly reduced IL-4, IL-5,TNF-α, leukotriene and total IgE levels as compared to baseline.^82^ Conversely, high concentrations of choline and the resulting elevation in TMAO plasma levels have been associated with increases in abundancy of TMA-producing bacteria (e.g. *Clostridiaceae* and *Coriobacteriaceae*) and the development of CVD and neurological disorders.

One of the mechanisms through which TMAO induces low-grade inflammation is the activation of NF-κB signaling. Seldin et al.^83^ demonstrated that Ldlr−/− mice fed a choline-rich diet showed elevated TMAO levels and a consequent increase in expression of pro-inflammatory genes including pro-inflammatory cytokines, adhesion molecules and chemokines in the aortic endothelium and smooth muscle cells. Through pharmacological inhibition, they showed that NF-κB and GPCR signaling are required for TMAO to exert pro-inflammatory effects on endothelial cells.

Another way through which TMAO exerts pro-inflammatory effects is activation of the inflammasome. In colon epithelial cells, TMAO was shown to trigger activation of the inflammasome and production of reactive oxygen species in a dose- and time-dependent manner, suggesting a potential role for TMAO in IBD pathogenesis.^21^ However, more research is needed in this context, as Wilson and colleagues found lower TMAO levels in IBD patients compared with healthy individuals.^84^ Similarly, they reported lower TMAO levels in patients with active UC compared to individuals with the inactive form of the disease.

Although the role of TMAO in the CNS has yet to be fully explored, it has recently been shown that TMAO can be detected in human cerebrospinal fluid. In addition, recent findings in mouse models have reported that TMAO promotes brain aging and cognitive impairment, as well as impaired long-term potentiation and synaptic plasticity in mice.^85^ In mice, TMAO concentrations in plasma and the brain were shown to increase in parallel with aging, suggesting that TMAO crosses the blood-brain barrier.^86^ The increased concentration of TMAO in whole-brain lysates of old mice was associated with higher brain pro-inflammatory cytokines (IL1-β, IL6, and TNF-α), total NF-κB abundance, and markers of astrocyte activation in comparison with young adult mice. Old mice also tended to have (presumably compensatory) higher brain anti-inflammatory IL-10 compared to young mice.^85^

In conclusion, these data indicate a strong link between microbiota-dependent production of TMAO, inflammation and an increased risk of, among others, cardiovascular and neurological disorders. Despite the overwhelming amount of evidence demonstrating correlations between TMAO, inflammation and CVD, these correlations are yet to be (fully) explored in other NCDs.^87^

## Bile acids

Bile acids (BAs) are a diverse group of amphipathic steroid acids derived from cholesterol in the liver.^88^ Apart from their essential role in vitamin absorption, lipid absorption, and cholesterol homeostasis, BAs have recently been recognized as important signaling metabolites in the regulation of the immune system.^28^ Two major classes of BAs exist in mammals: primary and secondary. Whereas primary BAs are synthesized via cholesterol catabolism in hepatocytes, secondary BAs are derivatives of primary BAs generated by microbial metabolism in the intestine.^89^ Primary BAs such as cholic acid and chenodeoxycholic acid (CDCA) are conjugated with glycine or taurine (in a lesser extent) in hepatocytes before their secretion into the intestinal environment. Deconjugation of BAs in the distal small intestine and colon is carried out by bacteria with bile salt hydrolase (BSH) activity such as *Lactobacillus, Bifidobacteria, Clostridium*, and *Bacteroides*. Deconjugated BAs then undergo different microbial biotransformations such as dehydrogenation, dihydroxylation, and epimerization in the colon leading to more than 20 different secondary BAs.^90,91^

BAs are ligands for nuclear hormone receptors such as the farnesoid X receptor (FXR) and GPCRs, such as TGR5, which inhibit the induction of pro-inflammatory genes through inhibition of the NF-ƙB pathway.^88,92^ Secondary BAs such as deoxycholic acid (DCA) and lithocholic acid (LCA) can suppress TNF-α production by macrophages via FXR. In the study by Renga et al.^93^ FXR−/− mice (FXR deficiency) exhibited an increase in colonic inflammation and cancer risk in comparison to the wild type. Guo et al.^94^ demonstrated that among BAs, secondary BAs (especially LCA) had the most potent effect on TGR5 signaling by inhibiting IL-1β production in macrophages. Clinical trials suggest that the elevated level of IL-1β in the circulation system is a risk factor for the development of T2D, and antagonism of IL-1β might be a promising treatment strategy. Moreover, secondary BAs can block the activation of the inflammasome.^95^ Excessive activation of inflammasome has been reported in T2D, atherosclerosis, and Alzheimer’s disease.^94^

The effect of secondary BAs on the immune system depends on the type of secondary BAs, concentration, and the site of action. For example, a metagenomic study revealed that the abundance of BSH genes (*bsh*) was significantly reduced in the gut microbiome of a patient with IBD.^96^ These data are in line with previous evidence indicating increased fecal conjugated BAs and reduced secondary BAs in IBD patients.^97^ Inconsistent with these data are studies on Alzheimer’s disease, in which a reduction in primary BAs and an increase in secondary BAs was found in the serum of patients.^98^ Additionally, it was shown in a hepatic cancer mouse model that the capability of secondary BAs to down-regulate the expression of chemokines was necessary for the accumulation of natural killer T cells. Inhibiting the accumulation of natural killer cells in the liver resulted in increased tumor growth.^99^ This inconsistency in the level of secondary BAs in different diseases might be the result of the activation of different BAs pathways.

Gut bacteria such as *Ruminococcus* can form ursodeoxycholic acid (UDCA), a secondary BA. UDCA can modulate the immune system through different mechanisms such as reduction of cytokine secretion by lymphocytes, immunoglobulin production, and inhibition of eosinophil activation and degranulation. UDCA exerts anti-inflammatory effects in the lungs. In an asthma mouse model, UDCA reduced airway inflammation via DCs by secretion of IL-12. Moreover, UDCA has dose-dependent (50–200 µM) neuroprotective effects by suppressing the NF-κB pathway.^100^ A growing number of pre-clinical and clinical studies underscore the potential benefit of UDCA in treatment of neurodegenerative diseases.^101^

The gut BAs pool modulates an important population of colonic Treg cells, the generation of which is defined by the expression of the transcription factor Foxp3. Genetic abolition of BAs metabolic pathways in individual gut symbionts significantly reduced the number of Foxp3^+^ Tregs, whereas restoration of the BAs pool increased the levels of this Treg population and alleviated host susceptibility to inflammatory colitis.^102^ Emerging studies have begun to unravel the molecular mechanisms underlying the regulation of colonic Tregs by the host-microbe biliary network. The secondary BA 3β-hydroxydeoxycholic acid (isoDCA) has recently been shown to induce Foxp3 in DCs, thereby impairing their immunostimulatory properties. Ablation of FXR in DCs potentiated the generation of Treg cells and imposed a transcriptional profile similar to that induced by isoDCA, suggesting an interaction between this BA and nuclear receptor.^103^ In addition, a derivative of LCA, isoalloLCA, was shown to promote Treg differentiation through the generation of mitochondrial reactive oxygen species, leading to increased Foxp3 expression.^104^

Overall, these data show that BAs have a pleiotropic signaling behavior in regulating the immune system and inflammation. Although much remains to be learned about the multifaceted functions of BAs *in vivo*, existing data already paint a picture that BAs have an important and dynamic role in controlling inflammation in NCDs.

## *N*-acyl amides

The interest in *N*-acyl amides has recently increased, especially with regard to their effect on the immune system. *N*-acyl amides such as *N*-arachidonoyl ethanol amide (anandamide), *N*-acyl glycine and *N*-acyl gamma amino butyric acid (*N*-acyl GABA) constitute an important family of endogenous lipid signaling molecules that are composed of a long-chain fatty acid connected to amino acids by an amide bond. *N*-acyl amides have similar effect on endogenous endocannabinoid receptors and are therefore considered as endocannabinoid analogs.^105^ In the last decades, research shed light on the importance of the endocannabinoid network in human physiology. The role of endocannabinoids in immune hemostasis has been hypothesized in different studies.^106,107^ However, the function of the endocannabinoid receptors on immune cells needs yet to be fully defined mechanistically. Endocannabinoids exert their actions mainly through GPCRs such as GPCR119, cannabinoid receptor (CB) 1, and CB2.^105,107^ They modulate T- and B-lymphocyte proliferation, macrophage-mediated killing of sensitized cells, inflammatory cytokine production, immune cell activation by inflammatory stimuli, chemotaxis, and inflammatory cell migration.^92^ However, there are a number of studies that show the double-edged sword effect of endocannabinoids as both inhibitor and stimulator of the immune system.^106^ For example, some preclinical studies revealed that the activation of CB1 might lead to CNS inflammation in neurodegenerative diseases.^108^ By contrast, some other studies showed that over-activation of CB1 contributes to symptom progression in Alzheimer’s disease and T2D.^109,110^ Hence, the effect of endocannabinoids may depend on different factors such as their concentration, target cells, and cellular environment.^111^ Gut microbiota can produce a large and diverse class of long-chain *N*-acyl amides that interact with endocannabinoid receptors, especially at the intestinal mucosa. Aguilera et al.^112^ showed that gut microbiota dysbiosis upregulated CB2 expression, which was positively correlated with *Lactobacillus* spp. counts and negatively correlated with C*lostridium* spp. counts. Moreover, a decreased amount of *Akkermansia muciniphila* has been reported in T2D patients (Table 1). Recently, it was shown that blocking CB1 ameliorates dysbiosis in T2D by increasing *A. muciniphila* abundance, which resulted in reduced inflammation.^113^ Cohen et al.^105^ reported the presence of 143 unique *n- acyl* genes in the human gut microbiota. Their products were classified into six different families: long-chain *N*-acyl- glycine, lysine, glutamine, lysine/ornithine, alanine, and serinol. Among all, N -palmitoyl serinol has been found as the strongest agonist for GPCR119. *N*-acyl amides were shown to prevent the cytokine-induced increase in gut permeability in an animal model of multiple sclerosis.^114^ Another study revealed that prolonged antibiotic treatment resulted in low levels of *N*-acyl serotonin in the intestine, which subsequently led to microglia inflammation and neuronal firing in depression mouse models.^115^ The mechanism of how gut dysbiosis affects the endocannabinoid network remains to be established. Future studies are required to spell out the distribution and concentration of these metabolites throughout the gastrointestinal tract.

In summary, the structural similarities of microbiota-encoded *N*-acyl amides and endogenous endocannabinoids suggest that gut microbiota might communicate with their host using the same chemical language. Production of diverse *N*-acyl amides by gut microbiota introduces this community as a potential source for endocannabinoid-based therapies. On the other hand, a new challenge arises to endocannabinoid system-based drugs, since microbial *N*-acyl amides may interfere with the normal function of endogenous endocannabinoid receptors and cause unwanted effects in non-target cells.^116^ As a dysregulated endocannabinoid network has been reported in different NCDs, understanding how gut microbiota contribute to this network is warranted and may suggest new therapeutic drugs for dysbiosis-related diseases.

## Vitamins

Another category of bacterial metabolites that are essential for host metabolism are vitamins. Microbial fermentation and modulation are essential for acquiring vitamin B and vitamin K, as the host is not capable to perform the necessary biosynthetic reactions.^40^ The members of the vitamin K group are mainly vitamin K1 (phylloquinone), and vitamin K2 (menaquinone) which are absorbed in the small intestine in a bile salt-, and pancreatic dependent solubilization.^117^ Vitamin K1 is obtained from ingested food and is mainly found in green leafy vegetables, while vitamin K2 is synthesized by certain intestinal bacteria, especially *Enterobacter* spp., *Eggerthella lenta, Veillonella* sp. and *Bacteroides* sp.^118,119^ Deficiency of vitamin K2 has been reported in several diseases such as cardiovascular, and neurodegenerative diseases, and the bacterial flora composition is significantly altered in patients suffering from these diseases.^120,121^ Besides the well-known role in blood coagulation, preventing osteoporosis and cardiovascular disease, recent studies indicate the significant effect of vitamin K on the immune system. Multiple studies showed that vitamin K2 is able to suppress the lipopolysaccharide-induced expression of inflammatory cytokines such as IL-6 by inhibiting the NF-κB pathway *in vitro*. Pan et al.^122^ showed that vitamin K2 was able to dose-dependently (10–100 μM) inhibit TNF-α, IL-1 α, and IL-1 β gene expression in human monocyte-derived macrophages *in vitro*. Moreover, vitamin K was shown to exert a protective effect against colitis through downregulation of the pro-inflammatory cytokine IL-6 in mice fed with a vitamin K-supplemented diet.^123^

Group B vitamins consist of essential micronutrients that are involved in multiple physiological processes to maintain the body’s homeostasis, such as lipid metabolism, erythrocyte formation, and cell division amongst others. These are water-soluble vitamins that are acquired from dietary sources as well as from our commensal gut bacteria which are both consumers and producers of vitamins B.^124^ *Lactobacilli* and *Bifidobacterium* are key producers of group B vitamins in the human intestine, being capable of synthesizing thiamine (B1), riboflavin (B2), niacin (B3), pantothenic acid (B5), pyridoxine (B6), biotin (B7), folate (B9), and cobalamin (B12).^124^ Recent studies indicate that B vitamins not only play an important role as cofactors and coenzymes in various metabolic pathways, but are also key to maintain immune homeostasis.^117^ Vitamin B deficiency increases the risk of developing immune-related diseases by inhibition of lymphocyte proliferation and suppression of natural killer cell activity.^125,126^ Thus, the production of vitamin B by the gut microbiota is essential in modulating host immune function.

Niacin, i.e. vitamin B3, has been shown to hamper inflammation by inhibiting the production of the pro-inflammatory cytokines IL-1, IL-6, and TNF-α by macrophages and monocytes.^127^ This anti-inflammatory effect of niacin was confirmed by Singh *et al*.^128^ who found that ligation of GPCR109a by niacin suppressed colonic inflammation and carcinogenesis by inducing T-regulatory cell differentiation in mouse models. In contrast, vitamin B5 stimulates pro-inflammatory effects, resulting in IL-6 and TNF-α production by macrophages and corresponding Th1 and Th17 responses.^129^ The mechanism underlying the regulation of inflammation by pyridoxine, i.e., vitamin B6, remains to be elucidated. However, high levels of IL-4 have been reported in individuals with vitamin B6 deficiency, thereby increasing the risk of developing inflammatory diseases such as allergy and rheumatoid arthritis.^130^ Biotin, i.e., vitamin B7, exhibits anti-inflammatory effects by suppressing NF-κB expression through histone biotinylation. It thereby prevents the production of several proinflammatory cytokines such as TNF-α, IL-1, IL-6, and IL-8.^131^ Biotin deficiency has been reported in different diseases such as IBD and chronic alcoholism. Suboptimal levels of biotin resulted in high levels of proinflammatory cytokines TNF-α, IL-1β, IL-12, and IL-23 in mouse models.^132^ As T-reg cells express high levels of folate receptor (folate receptor 4 [FR4]) it is suggested that folate, i.e. vitamin B9, exerts anti-inflammatory effects. Decreased cell viability was observed in T-reg cells cultured under vitamin B9-reduced conditions, whereas an increased susceptibility to intestinal inflammation was found in mice fed with a folate-deficient diet.^133^ At last, deficiency in cobalamin, i.e. vitamin B12, has been shown to decrease the number of CD8^+^ T cells and natural killer T cell activity in vitamin B12-deficient patients, whereas supplementation with methyl-cobalamin (known to be one of the strongest immunomodulators among the vit.B12 derivatives) ameliorated these conditions.^134^

Overall, vitamin K, as well as vitamin B, exert anti-inflammatory effects by downregulating the expression of pro-inflammatory genes. However, as is especially the case for vitamin B, the modulation of the immune system is specific to different immune cells and immune responses, evidenced by the involvement of different B vitamins in regulating different immune signaling pathways. Since not all bacteria within the intestinal microbiome possess a complete vitamin B synthesis pathway, competition may occur between the host and the intestinal microbiota for B vitamins as these bacteria utilize either dietary vitamin B or vitamin B produced by other bacteria. Research in this area remains challenging because vitamin-mediated regulation of immune homeostasis varies among individuals. This is not only due to inter-individual variability in gut microbiota composition but also due to the influence of poor dietary intake, nutrition and medication on intestinal microbiota diversity and functioning.

## Polyamines

Polyamines are a class of ubiquitous low-molecular-weight organic cations that are essential in various cellular processes such as proliferation, differentiation, and survival.^135^ Three polyamines, being putrescine, spermidine, and spermine, are part of the polyamine metabolic pathway that is critically regulated in a cell.^136^ Ingested food is the major source of polyamines in the upper parts of the intestine, from where they are transferred into the bloodstream via the colonic mucosa. As food-derived polyamines are absorbed before they reach the lower intestine, the gut microbiota is considered to be the major responsible cause of polyamine levels (0.5–1.0 mM) in the colon.^137,138^ The most abundant polyamines produced by bacteria are putrescine and spermidine which are precursors for other polyamines such as spermine. Spermine inhibits the synthesis of the pro-inflammatory cytokines mainly TNF-α, IL-1, IL-12, and IL-6.^139^ Moreover, spermine administration suppresses the progression of experimental autoimmune encephalitis, a model for multiple sclerosis, by inhibiting the pro-inflammatory activity of macrophages.^140^ Spermidine inhibits LPS-induced inflammation by blocking the NF-κB, and MAPKs signaling pathways in microglia. Nitric oxide and prostaglandin E2 production were decreased in a dose-dependent manner, as well as the mRNA expression of pro-inflammatory cytokines such as IL-6 and TNF-α.^141^ These data suggest that polyamines exert anti-inflammatory effects resulting in amelioration of various disease states. Although polyamine metabolism generates pro-inflammatory cytotoxic products such as hydrogen peroxide, research indicates that polyamines have mostly anti-inflammatory effects.^141^

In contrast to healthy tissues, a dramatic increase in polyamine levels is observed within cancerous tissues, as the activity of the rate-limiting enzyme for polyamine synthesis (i.e., ornithine decarboxylase) is upregulated by several oncogenic factors, including *MYC* family (regulatory gene and proto-oncogene).^142^ This allows the tumor cells to increase the uptake of polyamine from their microenvironment and thereby promotes tumor survival mechanisms by suppressing tumor-specific immune responses. This was underscored by Hayes et al.,^143^ who showed that polyamine blocking therapy combined with inhibiting polyamine transporters reversed the immunosuppressive environment in multiple murine tumor models.

Taken together, these data suggest that polyamines can have harmful as well as beneficial effects on human health in different disease states. Strategies to regulate and optimize the concentrations of polyamines in the intestinal tract are therefore likely to have practical applications.

### Profiling biochemical activity

**Figure 3.**
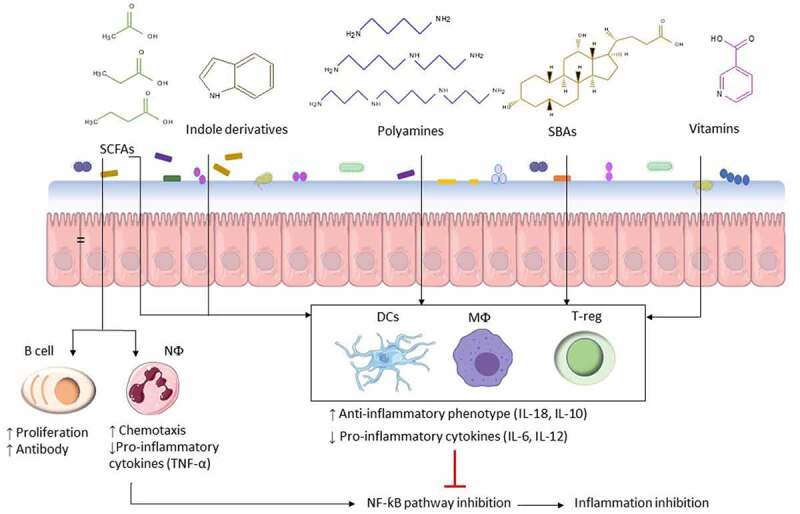
Anti- inflammatory and pro-inflammatory immune modulation by bacterial metabolites. Microbial-associated metabolites affect multiple facets of the immune response such as neutrophil (NФ) chemotaxis and cytokine secretion by macrophages (MФ), dendritic cells (DC), and T-regulatory cells (T-reg). Based on the immune response, secreted cytokines can suppress or activate the NF-κB pathway. NF-κB is a major transcription factor that regulates expression of pro-inflammatory genes responsible for both the innate and adaptive immune response and inflammation such as TNF-α, IL-6, IL-8, and IL-1β

Overall, many of the bacterial metabolites mentioned in this review affect certain immune signaling pathways such as NF-κB, which lead to down- or upregulation of pro-inflammatory factors (Figure 3). These effects make a ground for modulation of these immune pathways as a possible therapeutic target in NCDs. However, this review showed that unlike the involvement of certain receptors (e.g., HDACs, GPCRs, AHR and FXR), from a ligand point of view, a wide range of exogenous and endogenous metabolites can bind to these receptors and begin a cascade of immune signaling, indicating that the signaling function of metabolites may be overlapping. Therefore, two important questions need to be answered prior to any microbiota- mediated and/or metabolic pathway modulation.

1) How does modulation of metabolite concentration initiate inflammation and at what point does it become detrimental?

2) In which pathways is the targeted metabolite involved and what will be the system-level effect of this modulation, including the effect on the gut microbial population?

Answering these questions necessitates deep insight into dynamic modulation of biological processes. The power of metabolomics for dynamic measurement of phenotypes at the molecular level has brought this topic to the forefront of mechanistic discoveries related to pathophysiological processes. The recent advances in metabolomics methods such as untargeted metabolomics, isotope flux analysis, and high-resolution mass spectrometry techniques (liquid chromatography-mass spectrometry and gas chromatography-mass spectrometry) set the stage for more rigorous studies toward host-microbiota crosstalk.^32,144^

To fully comprehend causal interplay between the microbiota and the host immune system, a holistic understanding at all levels of the molecular architecture (genome, epigenome, transcriptome, proteome and metabolome) is needed. This requires an approach to connect complex datasets into a comprehensive model that enables a systematic analysis of their interactions and can propose novel hypotheses. To achieve this, computational systems biology approaches that integrate the different data types into a network model are necessary.^145^ Metabolomics is therefore of emerging interest for systems biologists as the metabolome represents a direct readout of the biochemical activity of the individual,^146^ and is therefore the most predictive of phenotype. The main advantage of metabolomics in systems biology is the connection of metabolic networks to the underlying reaction pathway structure thereby creating connectivity maps of pathways. Such reference map has recently been established for the human serum metabolome, in which potential determinants of more than 800 metabolites could be predicted. Using machine-learning algorithms (gradient-boosted decision trees), researchers were able to predict circulating blood metabolite levels in a healthy human cohort of 491 individuals based on host genetics, gut microbiome, clinical parameters, diet, lifestyle and anthropometric measurements. Metabolite levels were shown to be strongly affected by diet and microbiome, in some cases explaining more than 50% of the observed variance. Many of the associations and interactions found in this study could replicate previously reported findings, supporting the validity of this computational approach. The majority of them, however, are new, providing a useful resource for future research, either for unraveling a mechanistic understanding of alterations in metabolites under different conditions, or for designing interventions aimed at altering the levels of circulating metabolites.^3^

**Figure 4.**
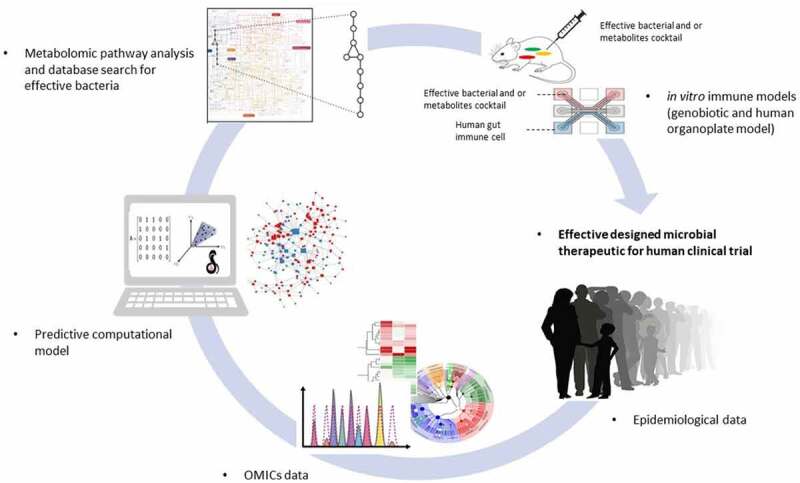
Integrated omics/systems biology/animal model approach to elucidate host-microbiota interactions in inflammation. Combining the results of the gnotobiotic and the metabolite- based approaches yields a set of bacteria capable of causally effecting an immune phenotype by potentially distinct mechanisms. These strains may be used together as rationally designed microbial therapeutics

Thus, systems biology-based metabolome profiling (Figure 4) can ultimately enable predictions of biomarkers and treatment response in a personalized manner and allow stratification of the patients as well as the proposition of potential novel therapeutic routes that can be validated experimentally and clinically.^4,146^ Systems biology approaches can be top-down or bottom-up. In the top-down approach, patterns and correlations are inferred from experimental data such as metabolomics. In the bottom-up approach, predictive computational models are generated by integrating different data types in a mechanistic manner.^4^ By integrating the microbiome, fecal metabolome, as well as microbial pathways and enzymes, links between microbial activity and changes in molecular architecture associated with NCDs (e.g., blood metabolome, transcriptome, proteome) could also be inferred. Integration of metabolomics with other -omics field such as metagenomics, transcriptomics, proteomics and culturomics will thereby hopefully lead to the systematic prediction and discovery of host-microbe metabolic interactions with a role in therapeutic outcomes.^5^

Using computer-guided design, Stein et al. have recently introduced a cocktail of 12 human-derived *Clostridia* as T-reg cell inducers due to their high SCFA outputs, which are currently undergoing clinical trials for the treatment of ulcerative colitis.^39^ Another study revealed a dysbiotic pattern in patients with Alzheimer’s diseases, while subjects without dementia or with other dementia types did not show this pattern.^6^ This pattern represented lower relative abundances of butyrate-producing species and higher relative abundances of taxa known to cause proinflammatory states in either neurological disorders or other colonic inflammatory states. These important advances bridge previous microbiome association studies with NCDs toward causality by showing how the dysbiotic microbiota can potentially adversely affect homeostasis via dysregulation of immune signaling pathways. These promising strategies are indispensable to support the conclusion that the relationship between the intestinal microbiota and an altered immune homeostasis is a means by which the microbiota impacts NCDs.

A variety of computational tools for the integration of multi-omics data is available.^7^ A recent devised computational approach, entailing the iterative coverage-based read assignment (ICRA) algorithm and the structural genomic variant finder (SGV-Finder), has been used to systematically identify microbial genomic structural variants across metagenomic samples of 887 healthy subjects. The ICRA algorithm resolves ambiguous read assignments to regions that are similar between different bacteria, after which the SGV-Finder systematically characterizes structural variation across ICRA-corrected reads in the healthy human microbiome. This approach enabled identification of SV-harbored genes with distinct functions associated with bacterial growth rates, as well as associations between SVs and host disease risk factors. For instance, SV-clustered genes in *Anaerostipes hadrus* encoded a composite inositol catabolism-butyrate biosynthesis pathway, the presence of which is associated with lower host metabolic disease risk. Overall, this novel computational modality exposes a new facet of the microbiome that aids in mechanistically understanding host–microbe interactions.^8^

One common bottom-up systems biology approach that enables data integration is constraint-based reconstruction and analysis (COBRA). COBRA relies on manually curated genome-scale reconstructions of the target organism that can readily be contextualized with omics data, e.g., transcriptomic or metabolomic data.^10^ Genome-scale reconstructions have been built for a number of organisms including human,^11^ mouse,^12^ rat,^13^ and 818 strains inhabiting the human gut.^14,124^ The latter resource, deemed AGORA, has been curated based on refined genome annotation and data from biochemical experiments and thus enables interrogating the metabolite biosynthetic capabilities of individual bacterial strains in the gut.^124^ By contextualizing AGORA with 16S rRNA or shotgun metagenomic data, the metabolic potential of a given microbiome can be mechanistically modeled.^124^ Such multi-species modeling approaches were applied to investigating host-microbe interactions linked to sulfide production in colorectal cancer,^16^ modeling the lung microbiome in Cystic Fibrosis patients,^19^ predicting dietary supplements that SCFAs production in Crohn’s Disease patients^147^ and analyzing microbial network patterns in relapsing Crohn’s Disease.^148^ In a recent study, the BA transformation potential of the microbiomes of Crohn’s Disease patients and healthy controls has been predicted and found to be distinct in the Crohn’s Disease microbiomes.^149^ In a follow-up effort, the metabolome of the same microbiomes was systematically predicted and also found to differ between dysbiotic and control microbiomes.^150^ The predicted metabolite fluxes have also been linked to the synthesizing strains and compared with published metabolomics data from the same cohort.^150^ Thus, these computational models can lead to mechanistic hypotheses linking altered bacteria to metabolites in NCD states.^151^

Software tools^152^ have been developed that enable deriving condition-and tissue-specific human cell models, e.g., of immune cells, from the human reconstruction,^153^ which could be subsequently integrated into microbiome modeling. That way, the effect of microbial metabolism on the host immune system could be interrogated specifically. Moreover, a whole-body model of human metabolism is available, which has been integrated with contextualized microbiome models resulting in personalized human-microbiome models.^154^ Candidate metabolites and relevant producer bacteria for a given immunomodulatory phenotype can be validated later by, for example, gnotobiotic or metabolic engineering studies to identify the functionally redundant group of commensal organisms.

### Conclusion

Research of the last decades indicated that metabolites secreted by the intestinal microbiota are undeniably involved physiological processes that are key for human health. Alteration in these bacterial metabolites during dysbiosis contributes to the downstream modulation of host immune homeostasis and chronic inflammation, a resemblance point in most NCDs. Therefore, identification of the bacterial metabolites that are involved in modulating the host immune response and the mechanism behind the modulation opens the door to the development of targeted microbial therapeutics efficacy via as-of-yet undiscovered mechanisms. The systems biology cycle of alternating experimental and computational approaches^4^ will be an indispensable tool for the elucidation of such mechanisms. Namely, an approach combining omics data generated from cohort studies, systems biology models, and *in vitro* (culture based) and animal models could lead to the identification of such mechanisms. For example, metabolic models could be contextualized with omics data and used to predict mechanisms for potential host-microbiome-disease interactions. These mechanisms could then be validated in *in vitro* and animal experiments.

Combination of metabolomics with other -omics, bioinformatics, and systems biology approaches allow us to cope with most of the complications described in the present article, and gives clinically and pharmacologically meaningful outcomes. For example, trans-omics strategy helps pharmaceutical research to look at this field as a challenging and exciting path to treat disease such as NCDs. It has been suggested that therapeutic and dietary interventions should be patient-specific.^155^ By integrating omics data with pharmacokinetic and metabolic modeling, personalized models could be created, which could lead to the prediction of such patient-specific drug- and dietary interventions and subsequent validation.
